# Matrix Metalloproteinase-9 Production following Cardiopulmonary Bypass Was Not Associated with Pulmonary Dysfunction after Cardiac Surgery

**DOI:** 10.1155/2015/341740

**Published:** 2015-07-27

**Authors:** Tso-Chou Lin, Feng-Yen Lin, Yi-Wen Lin, Che-Hao Hsu, Go-Shine Huang, Zhi-Fu Wu, Yi-Ting Tsai, Chih-Yuan Lin, Chi-Yuan Li, Chien-Sung Tsai

**Affiliations:** ^1^Department of Anesthesiology, Tri-Service General Hospital, National Defense Medical Center, Taipei, Taiwan; ^2^Department of Internal Medicine, School of Medicine, College of Medicine, Taipei Medical University, Taipei, Taiwan; ^3^Division of Cardiology, Department of Internal Medicine, Taipei Medical University Hospital, Taipei, Taiwan; ^4^Division of Cardiovascular Surgery, Department of Surgery, Tri-Service General Hospital, National Defense Medical Center, Taipei, Taiwan; ^5^Graduate Institute of Clinical Medical Sciences, China Medical University, Taichung, Taiwan; ^6^Department of Anesthesiology, China Medical University Hospital, Taichung, Taiwan

## Abstract

*Background*. Cardiopulmonary bypass (CPB) causes release of matrix metalloproteinase- (MMP-) 9, contributing to pulmonary infiltration and dysfunction. The aims were to investigate MMP-9 production and associated perioperative variables and oxygenation following CPB. *Methods*. Thirty patients undergoing elective cardiac surgery were included. Arterial blood was sampled at 6 sequential points (before anesthesia induction, before CPB and at 2, 4, 6, and 24 h after beginning CPB) for plasma MMP-9 concentrations by ELISA. The perioperative laboratory data and variables, including bypass time, PaO_2_/FiO_2_, and extubation time, were also recorded. *Results*. The plasma MMP-9 concentrations significantly elevated at 2–6 h after beginning CPB (*P* < 0.001) and returned to the preanesthesia level at 24 h (*P* = 0.23), with predominant neutrophil counts after surgery (*P* < 0.001). The plasma MMP-9 levels at 4 and 6 h were not correlated with prolonged CPB time and displayed no association with postoperative PaO_2_/FiO_2_, regardless of reduced ratio from preoperative 342.9 ± 81.2 to postoperative 207.3 ± 121.3 mmHg (*P* < 0.001). *Conclusion*. Elective cardiac surgery with CPB induced short-term elevation of plasma MMP-9 concentrations within 24 hours, however, without significant correlation with CPB time and postoperative pulmonary dysfunction, despite predominantly increased neutrophils and reduced oxygenation.

## 1. Introduction

Cardiopulmonary bypass (CPB) induces a systemic inflammatory response [1] and the following postoperative pulmonary dysfunction [2]. After initiating CPB, blood cells are activated by contact with artificial surfaces of circuits and direct contact of air and damaged tissues, resulting in release of various proinflammatory cytokines. The cessation of alveolar ventilation and ischemia-reperfusion injury leads to neutrophil activation [3] and trafficking to lung [4] and entrapment in the pulmonary capillaries. The released proteolytic enzymes, such as matrix metalloproteinase- (MMP-) 9, degrade alveolar basement membrane and matrix [5], facilitating neutrophil transmigration and protein extravasation into the interstitial tissue of lung [6]. Consequently, injury to the alveolar endothelium and alveolar-capillary barrier dysfunction as central events in the pathogenesis of acute lung injury [7] are manifested with postoperative pulmonary edema and abnormal gas exchange after cardiac surgery with CPB.

The MMPs are a family of more than 25 species of zinc-dependent proteases that are essential for normal tissue remodeling in processes including bone growth, wound healing, and reproduction [5]. Among them, elevated MMP-9 concentration has been a risk factor for future myocardial infarction [8] or coronary revascularization [9] and heart failure [10] by myocardial remodeling [11] after acute myocardial infarction, in addition to acute lung injury [12] and chronic obstructive pulmonary disease [13]. Some clinical studies demonstrated increased MMP-9 levels following CPB for cardiac surgery [14, 15], however, with scarce evidence of correlation with postoperative pulmonary dysfunction, except in rat models with CPB-induced [16] or pancreatitis-associated [17] lung injury. We therefore hypothesized that MMP-9 activation following CPB may contribute to postoperative pulmonary dysfunction. The aims of this study were to investigate MMP-9 concentrations following CPB and analyze the association with perioperative variables and postoperative pulmonary parameters PaO_2_/FiO_2_ among patients undergoing elective cardiac surgery.

## 2. Materials and Methods

### 2.1. Participants

The study was approved by the ethics committee (TSGHIRB-1-101-05-115) and written informed consent was obtained from 30 patients undergoing elective cardiac surgery. Patients with cancers, patients with autoimmune diseases, patients receiving steroids, and patients with preoperative respiratory or hepatic failure were excluded.

### 2.2. Perioperative Management

In the operation room, patients were premedicated with fentanyl and midazolam for arterial catheterization. General anesthesia was induced with fentanyl 1.5–3 *μ*g/kg, propofol 0.5–1.5 mg/kg, and cisatracurium 0.1–0.2 mg/kg and maintained with sevoflurane or isoflurane after tracheal intubation. A pulmonary artery catheter was placed through right internal jugular vein and transesophageal echocardiography was used to monitor real-time cardiac performance throughout the whole procedure. Routine median sternotomy and standard cardiopulmonary bypass (Sarns 8000, Terumo, Ann Arbor, MI) with an extracorporeal membrane oxygenator (Capiox SX 18, Terumo, Ann Arbor, MI) were carried out in sequence to maintain the body temperature at 28–30°C during surgery. The perfusionist adjusted sevoflurane or isoflurane concentration on the vaporizer to keep mean arterial blood pressure between 50 and 80 mmHg during bypass period. Following standard rewarming and deairing, the pump was weaned at the end of the procedure. Routine inotropic support including dopamine or dobutamine infusion was added for acceptable cardiac output, if necessary. All patients were transferred to the cardiovascular surgical ICU with endotracheal intubation after surgery.

### 2.3. Blood Samples and Data Collection

Each 20 mL of arterial blood was sampled from the arterial line at 6 sequential time points (before induction, just before CPB, and at 2 h, 4 h, 6 h, and 24 h after beginning CPB) into EDTA-containing tubes. After immediate centrifugation of blood samples at speed 1500 rpm for 10 min at room temperature, the plasma samples were divided into Eppendorf tubes and frozen at −70°C for later plasma MMP-9 concentration testing.

Arterial blood gas analysis was routinely examined perioperatively and immediately upon ICU admission by GEM Premier 3000 (Instrumentation Laboratory, Lexington, MA). Perioperative variables, including general anesthesia, operation, and bypass times, as well as preoperative and postoperative laboratory data, were also recorded. The ratio of arterial oxygen tension (PaO_2_, expressed in mmHg) to inspired oxygen fraction (FiO_2_) was also calculated before and after operation, as well as before extubation. The severity of hypoxemia is defined as mild (200 < PaO_2_/FiO_2_ ≤ 300 mmHg), moderate (100 < PaO_2_/FiO_2_ ≤ 200 mmHg), and severe (PaO_2_/FiO_2_ ≤ 100 mmHg) by the Berlin definition of acute respiratory distress syndrome [18].

### 2.4. Enzyme-Linked Immunosorbent Assay (ELISA)

The protein concentrations of MMP-9 in the plasma were measured by using the commercially available DuoSet ELISA development kits (R&D Systems Inc., McKinley Place N.E., Minneapolis, USA) according to the manufacturer's protocol (MMP9 catalog number DY911). The absorbance of the color at 450 nm was recorded using a TECAN Sunrise ELISA Reader (Tecan Group Ltd., Männedorf, Switzerland).

### 2.5. Statistical Analysis

The results were analyzed through SPSS software version 17 (SPSS, Chicago, IL). The demographic data, perioperative variables, postoperative leukocyte counts, arterial blood gas data, and plasma MMP-9 concentrations were presented as mean ± SD. The correlation between MMP-9 concentrations, CPB time, and pulmonary PaO_2_/FiO_2_ ratios was analyzed by paired *t*-test or Pearson correlation analysis. *P* value <0.05 was considered statistically significant.

## 3. Results

Demographic data of 30 patients were summarized in Table 1, with mean CPB time 120.8 ± 52.4 min. The postoperative white blood cell and neutrophil counts increased significantly (Table 2), whereas lymphocyte and platelet counts decreased, as compared with the preoperative data (*P* < 0.001). The blood glucose levels were significantly elevated from 135.7 ± 37.1 (range 83–221) to 210.2 ± 44.1 (range 126–338) mg/dL postoperatively (*P* < 0.001). To examine alveolar oxygen exchange, PaO_2_/FiO_2_ ratio was calculated. There were only 12 patients having a preoperative ratio within 200–300 mmHg (mild hypoxemia), while turning to 3 patients with ratio ≤100 mmHg (severe hypoxemia), 16 within 100–200 mmHg (moderate hypoxemia), and 6 within 200–300 mmHg (mild hypoxemia) postoperatively. The mean PaO_2_/FiO_2_ ratio reduced from preoperative 342.9 ± 81.2 (range 232.0–563.0) to postoperative 207.3 ± 121.3 (range 67.0–538.3) mmHg (*P* < 0.001) (*n* = 30) but recovered before extubation 325.7 ± 129.8 (range 156.0–597.5) mmHg (*P* = 0.456), with extubation time 38.0 ± 28.6 (range 3.5–140) hours after arriving at ICU (*n* = 27), except for 3 patients with pneumonia or mediastinitis-related septic shock, and expired at postoperative 10, 25, and 31 days in the ICU. The mean ICU stay (*n* = 27) was 3.5 ± 1.4 (range 2–9) days, with mean hospital stay 11.7 ± 3.9 (range 7–23) days after surgery.

**Figure 1:**
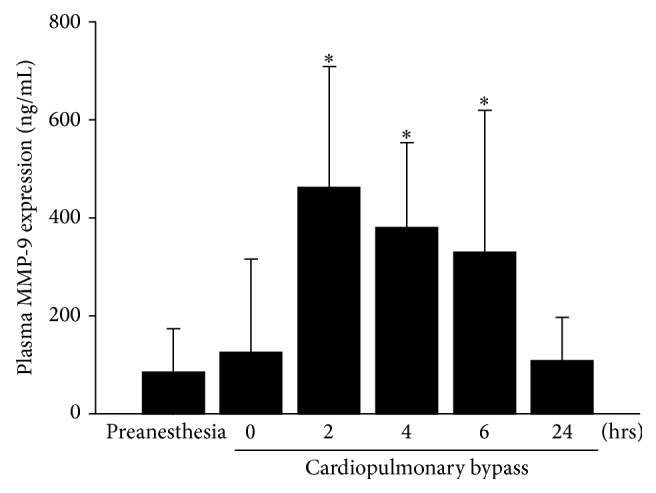
Plasma matrix metalloproteinase-9 concentrations increased significantly at 2–6 hours after beginning cardiopulmonary bypass (*n* = 30), as compared with the preanesthesia level (all *P* < 0.001). The level returned closely to the preanesthesia level at 24 hours (*P* = 0.23).

As shown in Figure 1, the plasma MMP-9 levels (*n* = 30) rose significantly at 2, 4, and 6 hours after the start of CPB, with 462.6 ± 247.2 (range 65.5–1102.4), 381.1 ± 174.3 (range 144.1–843.8), and 331.4 ± 288.4 (range 19.9–1266.4) ng/mL, respectively, as compared with 85.7 ± 88.8 ng/mL before anesthesia induction (all *P* < 0.001). The mean level returned closely to the preanesthesia value at 24 hours after the start of CPB (109.3 ± 88.6 ng/mL, *P* = 0.23).

Using Pearson correlation analysis, plasma MMP-9 concentrations at 4 and 6 hours after initiation of CPB were not correlated with CPB time (*P* = 0.60 and 0.83, resp.). Besides, the MMP-9 levels at 4 hours displayed no differences between patients with less or more than 121 min CPB time (376.6 ± 193.3 versus 388.8 ± 143.9 ng/mL, *P* = 0.86). To test the MMP-9 effect on pulmonary dysfunction, the MMP-9 levels at 4 and 6 hours were not associated with postoperative PaO_2_/FiO_2_ ratio (*P* = 0.63 and 0.48, resp.) and neither was CPB time for PaO_2_/FiO_2_ (*P* = 0.25).

## 4. Discussion

In this study, elective cardiac surgery with CPB induced a transient elevation of MMP-9 concentrations at 2–6 hours after beginning CPB, with a mean CPB time of 120.8 minutes. However, the MMP-9 levels at 4 and 6 hours were not correlated with the bypass time and postoperative PaO_2_/FiO_2_ ratio, despite predominant increase of neutrophil counts upon arriving at ICU, mostly at 4 hours after beginning CPB.

CPB causes a systemic inflammatory response, including activation of neutrophils [3], which are chemoattracted to the inflammatory site. Degranulation of the inflammatory mediators from neutrophils, such as reactive oxygen species and proinflammatory cytokines, could cause pulmonary dysfunction after cardiac surgery by augmenting both neutrophil-pulmonary endothelial adhesion and change of alveolar-endothelial permeability [19]. Also, MMP-9 is degranulated from neutrophils to degrade type IV collagen, the major constituent of basement membrane, and to facilitate neutrophil extravasation. In a canine myocardial ischemia/reperfusion model, infiltrating neutrophils are an early source of MMP-9 after reperfusion [20]. Our previous study [14] demonstrated that intracellular MMP-9 protein and mRNA expression of neutrophils increased after beginning CPB, consistent with the increase of plasma MMP-9 concentrations during cardiac surgery. In this study, we demonstrated a similar trend of MMP-9 production following cardiac surgery and tried to analyze the correlation between MMP-9 concentration and CPB time. With a mean 2-hour bypass period, most cardiac operations in this study were finished 2 hours thereafter. Therefore, blood samples obtained upon arriving at the ICU (mostly 4–6 hours after beginning CPB) could be an indicator for ongoing MMP-9 overproduction or remission after surgery. Among our patients, the MMP-9 concentrations at 4–6 hours were not correlated with CPB time, indicating rapid remission of MMP-9 production after elective cardiac surgery with relative shorter CPB time, despite significant increase of neutrophil counts after surgery.

Cardiac surgery using CPB may cause postoperative pulmonary dysfunction, including acute lung injury and/or acute respiratory distress syndrome (ARDS) [2]. According to the 2012 Berlin definition of ARDS [18], the severity of pulmonary dysfunction is updated by degree of hypoxemia as mild (200 < PaO_2_/FiO_2_ ≤ 300 mmHg), moderate (100 < PaO_2_/FiO_2_ ≤ 200 mmHg), and severe (PaO_2_/FiO_2_ ≤ 100 mmHg). In our patients, postoperative depression of oxygenation was observed, including reduced PaO_2_/FiO_2_ ratio and patients shifting from preoperative mild hypoxemia (*n* = 12) to postoperative severe (*n* = 3), moderate (*n* = 16), and mild (*n* = 6) hypoxemia. Based on the previous evidence of predominant neutrophil recruitment and activation following CPB [6, 14], neutrophil-mediated MMP-9 activation [14, 20], and MMP-involved acute lung injury [7, 12], we hypothesized that MMP-9 activation may contribute to pulmonary dysfunction following CPB, which could be manifested by reduced PaO_2_/FiO_2_ ratio. Eventually, our results demonstrated transient enhancement of MMP-9 concentrations at 4–6 hours following CPB, which, however, was not correlated with reduced postoperative PaO_2_/FiO_2_, indicating clinically short-term and insignificant influence on pulmonary function in our patients with elective cardiac surgery.

Two limitations should be addressed. First, only the plasma MMP-9 concentrations and clinical parameters were analyzed during the acute phase in this study, needing further ex vivo or experimental data to verify the biological changes of acute lung injury and the following myocardial remodeling. Second, limited case number in this study may diminish the clinical manifestation and its significance of correlation. More participants with various groups of severity are needed to verify MMP-9 as a clinical indicator for pulmonary dysfunction after cardiac surgery.

In conclusion, we identified that elective cardiac surgery with CPB induced a short-term elevation of MMP-9 concentrations at 2–6 hours after beginning CPB, with predominant increase of neutrophils. The postoperative MMP-9 levels upon arriving at ICU were not correlated with bypass time and reduced oxygenation after cardiac surgery.

## Figures and Tables

**Table 1:** Demographic data and perioperative variables (*n* = 30).

Gender (male/female)	23/7	
Age, year	60.0 ± 10.5	(33–80)
Height, cm	163.9 ± 7.3	(148–179)
Weight, kg	68.1 ± 13.7	(43–104)
Body mass index, kg/cm^2^	25.3 ± 4.4	(17.2–37.3)
General anesthesia time, min	345.6 ± 75.1	(237–495)
Operation time, min	290.3 ± 67.3	(184–441)
Cardiopulmonary bypass time, min	120.8 ± 52.4	(49–281)
Aortic clamp time, min	75.2 ± 35.2	(16–167)
Ischemic heart disease	15	
Coronary artery graft	2.7 ± 0.6	(1–3)
Valvular heart disease	12	
Heart tumor	2	
Atrial septal defect	1	
Hypertension	20	
Diabetes mellitus	10	

The data were presented as mean ± SD (range).

**Table 2:** Perioperative laboratory data and variables.

	Preoperative	Postoperative	*P* value
White blood cell count, /mm^3^	7,500 ± 2,300	11,500 ± 4,000	<0.001
N ratio, %	65.4 ± 8.6	86.3 ± 5.5	<0.001
L ratio, %	22.9 ± 8.5	7.8 ± 3.2	<0.001
Neutrophil count, /mm^3^	5,000 ± 2,000	10,000 ± 3,700	<0.001
Lymphocyte count, /mm^3^	1,600 ± 600	800 ± 300	<0.001
Platelet, /mm^3^	229.8 ± 70.7	152.4 ± 38.7	<0.001
Hemoglobin, g/dL	13.0 ± 2.0	10.2 ± 1.5	<0.001
BUN, mg/dL	21.4 ± 14.5	18.2 ± 10.2	0.101
Creatinine, mg/dL	2.0 ± 2.9	1.6 ± 1.9	0.202
AST, U/L	29.9 ± 22.2	40.7 ± 16.8	0.002
ALT, U/L	29.0 ± 23.0	21.0 ± 8.7	0.027
Glucose, mg/dL	135.7 ± 37.1	210.2 ± 44.1	<0.001
Lactate, mmol/L	1.0 ± 0.4	3.2 ± 1.9	<0.001
Base excess, mmol/L	2.2 ± 1.9	0.1 ± 4.1	0.012
HCO_3_^−^, mmol/L	25.7 ± 2.2	25.2 ± 3.5	0.466
Troponin-I, ng/mL		3.7 ± 2.8	
B-type natriuretic peptide, pg/mL		213.1 ± 242.2	
Cardiac index, L/min/m^2^		3.3 ± 1.1	
PaO_2_/FiO_2_ ratio, mmHg	342.9 ± 81.2	207.3 ± 121.3	<0.001
PaO_2_/FiO_2_ ratio before extubation		325.7 ± 129.8	0.456^*^
Extubation time, hours		38.0 ± 28.6	
ICU stay, days		3.5 ± 1.4	
Hospital stay, days		11.7 ± 3.9 (7–23)	

^*^Compared with the preoperative data.
